# Do Future Limitation Perspective in Cancer Patients Predict Fear of Cancer Recurrence, Mental Distress, and the Ventromedial Prefrontal Cortex Activity?

**DOI:** 10.3389/fpsyg.2018.00420

**Published:** 2018-03-28

**Authors:** Jia Zhou, Pan Feng, Xiaofei Lu, Xingping Han, Yanli Yang, Jingjing Song, Guangyu Jiang, Yong Zheng

**Affiliations:** ^1^Center for Studies of Education and Psychology of Ethnic Minorities in Southwest China, Southwest University, Chongqing, China; ^2^School of Humanities and Management Science, Southwest Medical University, Luzhou, China; ^3^Faculty of Psychology, Southwest University, Chongqing, China; ^4^Department of Radiology, Affiliated Hospital of Southwest Medical University, Luzhou, China; ^5^Department of Oncology, Affiliated Hospital of Southwest Medical University, Luzhou, China

**Keywords:** cancer, future limitation perspective, fear of cancer recurrence, mental health status, resting-state fMRI, ventromedial prefrontal cortex

## Abstract

Life-threatening diseases (e.g., cancer) affect people’s future time perspective (FTP) and affect their mental health. When one’s lifetime is perceived as running out, the individual possesses a future limitation perspective (FLP), which is one of factors in FTP. In this study, we explored the structural relationship between FLP, fear of cancer recurrence (FCR), mental health status (MHS), and brain activity in patients with cancer. Cancer patients were divided into two groups using the FTP scale and Feelings About Life Scale: a strong FLP group (S-FLP) and a weak FLP group (W-FLP). For these groups, we measured cancer patients’ MHS using the Symptom Checklist (SCL-90) and FCR using the Cancer Acceptance Scale; brain activity was measured using resting-state functional magnetic resonance imaging (rs-fMRI). Behavioral results showed that the S-FLP group had higher mental symptoms and FCR scores than did the W-FLP group. Neuroimaging results revealed that spontaneous brain activity in the ventromedial prefrontal cortex (vmPFC) was stronger in the W-FLP group than in the S-FLP group. Moreover, brain activity in the vmPFC negatively correlated with FLP, FCR, and SCL-90 scores only in the S-FLP group, and the model constructed further indicated that FCR and SCL-90 scores fully mediated the relationship between FLP and vmPFC activities. These findings suggested that a strong FLP might lead to mental disorders and greater FCR, which might change the spontaneous activity of the vmPFC in cancer patients.

## Introduction

Statistics in China (Chen et al., 2016) have shown that 4,292,000 new cancer cases and 2,814,000 cancer deaths would occur in 2015. Although cancer is a serious illness, it may not lead to immediate death. The vast majority of cancer patients will experience a relatively long course of treatment, which brings considerable suffering. Throughout cancer treatment, cancer patients not only suffer from physical changes, but also psychological changes. An important psychological issue among patients with cancer is their perception of their own lifetime, or temporal extension perception. Temporal extension refers to the perceived length of one’s past, present, and future relative to one’s lifetime, and is an integral aspect of time perception (van Laarhoven et al., 2011). Future time perspective (FTP) is a future-oriented temporal extension referring to one’s remaining lifespan, often based on their current objective life situation (e.g., health state). FTP ranges on a continuum from opportunities to limitations (Cate and John, 2007). When one’s lifetime is perceived as running out, the individual is defined as possessing a future limitation perspective (FLP); in contrast, when their lifetime is perceived as open-ended (expansive), they are considered to have a future opportunities perspective (FOP). Therefore, cancer patients’ forming of their FTP is based on their disease state and treatment effects (Cunningham et al., 2002). Having greater health constraints and lower subjective health is associated with a stronger FLP (Grühn et al., 2015). Stronger FLP leads to death salience and the threat of death produces existential terror (Bergamini, 2013), and patients will experience more psychological distress (Lang and Carstensen, 2002; Pinquart and Silbereisen, 2006; Dousti et al., 2013), particularly related to their own mortality (terror-management theory) (Burke et al., 2010).

Under the influence of FLP, cancer patients tend to fear cancer recurrence or metastasis, which can lead to dysfunctional mental states (Clark et al., 1994). Patients in remission (Black and White, 2005) in particular may experience a fear of cancer recurrence (FCR), derived from a core view of cancer as a vicious, unpredictable, and indestructible enemy. Their fear tends to relate to the proximity of cancer, lack of strategies to keep it at bay, and fear of dying from cancer (D’Souza et al., 2016; Vrinten et al., 2016). Accordingly, FCR can be defined as the fear or worry that the disease will return or progress in the same organ or in another part of the body (Vickberg, 2003). The FCR is often so overwhelming that it influences cancer patients’ quality of life (Custers et al., 2014). FCR can lead to persistent dysfunctional behaviors, including avoidance, over-vigilance for symptoms of recurrence (Mcginty et al., 2008), and an inability to plan for the future (Pinquart and Silbereisen, 2006). In patients with severe FCR, FCR has been associated with the development of anxiety disorders, depression, and post-traumatic stress symptoms (Black and White, 2005; Rauch et al., 2006; Twigg et al., 2008).

Both FLP and FCR can lead to disorders in the cancer patient’s mental health. These cancer patients exhibit poorer spontaneous adjustment ability in the psychological issues. This is similar to problems observed in individuals with lifetime post-traumatic stress symptoms (lifetime-PTSS) (Smith et al., 1999; Walker et al., 2003; Black and White, 2005). The pathogenesis of lifetime-PTSS can be conceptualized as a fear process: the disease poses a threat to life and therefore activates fears of life being shortened, along with fears of failure of treatment during the course of treatment (Rauch et al., 2006). Neuroimaging research has implicated that lifetime-PTSS is associated with hypo-activation of the ventromedial prefrontal cortex (vmPFC) and hyper-activation in the amygdala and other regions. The vmPFC serves a key role in emotional regulation and behavioral control (Etkin et al., 2011). Some research on the neural correlates of time perspective has indicated that the vmPFC is positively activated during future-positive thinking (Bölter et al., 2010). However, no studies have examined how brain activities change as per the altering mental states among cancer patients with a negative future perspective. In particular, it is not clear how the enormous limitation caused owing to the pressure of survival (i.e., FLP) and disease-related fears (i.e., FCR) correlated to the mental-health influence brain functions in cancer patients.

In this study, we adopted an important neuroimaging tool called resting-state functional magnetic resonance imaging (rs-fMRI), which can measure intrinsic spontaneous brain activity (Fox and Raichle, 2007). Specifically, the amplitude of low-frequency fluctuations (ALFF) was calculated and used as an index of regional spontaneous neuronal activity in blood-oxygen-level dependent (BOLD) signals (Zou et al., 2008). Based on prior research, we expected that FTP would increase FCR and lead to worse mental health. Moreover, we further examined the differences in spontaneous activity between a strong FLP (S-FLP) group and a weak FLP (W-FLP) group, as well as the association among FLP, FCR, mental health status (MHS), and spontaneous activity. The specific hypotheses of this study were as follows: (a) following a cancer diagnosis, a stronger FLP will be linearly associated with greater FCR and (b) worse MHS. Furthermore, (c) greater FCR will be associated with greater deterioration in MHS, and (d) stronger FLP, greater FCR, and worse MHS will lead to changes in brain activity.

## Materials and Methods

### Participants

At the beginning of this study, we recruited 140 participants from the department of oncology. Eligible participants had a clinical diagnosis of any cancer and were aware of their disease status. Each potential participant completed the limitation subscales of the Future Time Perspective Scale (Lang and Carstensen, 2002) and Feelings About Life Scale (Cate and John, 2007). The scores of these two subscales were summed and used as the grouping criteria. In order to match the numbers of the two groups, we chose 55 participants whose total score of the questionnaire ranked from high to low, and then we chose 55 participants whose total score of the questionnaire ranked from low to high. For both groups, the exclusion criteria for patients were as follows: (1) experienced recurrence or metastasis; (2) had tumors originating in or metastasized to the head; (3) treated for cancer for less than a year; and (4) meeted the criteria for brain imaging data. Finally, we got the high-score group (45 cancer patients) which was the S-FLP group, and low-score group (49 cancer patients) which was the (W-FLP) group.

In total, 94 cancer patients participated in the study (age range = 20–79 years, *M*_age_ = 50.31, *SD* = 12.34, 55 females), with 45 in the S-FLP group (*M*_age_ = 51.64, *SD* = 12.68, 27 females) and 49 in the W-FLP group (*M*_age_ = 49.08, *SD* = 12.01, 28 females). No significant group differences were found in age (*t*[92] = 0.32, *p* = 1.01), gender (χ^2^(1) = 0.79, *p* = 0.78), or the time interval between becoming aware of the cancer diagnosis and carrying out this study (*M*_S-FLP_time_interval_ = 13.87 months, *SD* = 17.95, *M*_W-FLP_time_interval_ = 16.17 months, *SD* = 17.95, *t*[92] = -0.57, *p* = 0.56).

### Ethics Statement

This study was approved by the Institutional Review Board of Southwest University and the Affiliated Hospital of Southwest Medical University and the reference number is XNYD2017268. Written informed consent was obtained from all participants after a complete description of the study procedure to them. After completing the experiments, we provided participants with a gift (a set of bowls valued at 20 Yuan RMB) and a monetary reward (200 Yuan RMB) to express our gratitude for their participation.

### Procedure

The experiment consisted of two sessions: questionnaires and the rs-fMRI scans. It took about 2 h for the patient to complete both the parts. To prevent fatigue, these experiments were conducted over 2 days and not necessarily for 2 h continuously. All patients were recruited while receiving inpatient care. During interviews with the patients, the physicians invited the patients to answer the study questionnaires (three questionnaires in total) and participated in the rs-fMRI scans. During fMRI scanning, participants were instructed to relax, keep their eyes closed, stay awake, remain still, and not think of anything in particular. The scan lasted for 14 min and included two parts: T1-weighted images and echo-planar imaging (EPI) sequence. The resting state scan lasted for 300 s, and a total of 150 functional volumes were acquired.

### Measures

#### Future Limitation Perspective

Our measure of FLP included items from two sources: the FTP scale (Lang and Carstensen, 2002) and the FAL scale. The FTP scale comprises two subscales, including opportunities and limitations. In the present study, we focused on the limitation subscale items, and its Cronbach’s alpha was 0.800. We revised one item “As I get older, I begin to feel that time is limited” into “Since I got this disease, I have begun to feel that time is limited” to fit our study purposes. The FAL scale (Cate and John, 2007) also includes subscales of opportunities and limitations, and we again focused on the limitation factor, and its Cronbach’s alpha was 0.831. Each item in both scales is rated on a five-point Likert scale, ranging from 1 (extremely disagree) to 5 (extremely agree). We summed the scores of both limitation subscales to measure patients’ FLP and screen out the S-FLP and W-FLP groups, as noted above. This FLP scale had good reliability and validity in the present sample. Specifically, the Cronbach’s alpha was 0.902. Furthermore, the exploratory factor analysis revealed one component explaining 68.1% of the total variance. The Kaiser–Meyer–Olkin measure of sampling adequacy was 0.901, and Bartlett’s test of sphericity was significant (χ^2^ = 320.73, *df* = 15, *p* = 0.000).

#### Fear of Cancer Recurrence

The modified version of the Cancer Acceptance Scale (CAS) (Servaes et al., 2003; Gielissen et al., 2006) assesses a patient’s fear of disease recurrence and comprises two items. Both items are rated on a four-point Likert scale ranging from ‘does not apply to me at all’ to ‘completely applies to me,’ and total scores range from 2 to 8. The Cronbach’s α was 0.85 in the present sample.

#### Mental Health Status

This measurement consists of two parts. In first part, the cancer patients were asked: “Is mental health before diagnosis of cancer was in a good status?” to assess the changes of cancer patients’ MHS with or without cancer. Participants responded “Yes” or “No.”

In the second part, we used the Symptom Checklist-90 Revised (SCL-90-R) (Derogatis, 1996) to measure the extent and severity of psychological symptoms of a person. It has a good potential to identify people with psychological problems, either at the subthreshold or clinical level. The scale comprises 90 items in 10 dimensions. Each dimension represents a single psychological symptom, and is rated on a five-point scale from 1 (do not have) to 5 (severe). The Cronbach’s α was 0.94 in the present sample.

#### MRI Acquisition

We used a Philips-Achieva-3.0 T MRI scanner to collect functional imaging data. Head movement was restricted using foam cushions. The resting-state functional images were recorded using Echo-planar imaging (EPI) sequences (TR = 2500 ms; TE = 30 ms; flip angle = 90°; slice thickness = 3.0 mm; FOV = 224 mm × 223 mm; acquisition matrix = 76 × 79; voxel size = 2.9 mm × 2.8 mm × 3 mm; interslice skip = 0.99 mm; slices = 52). The T1-weighted images were also recorded with a total of 220 slices at a thickness of 1 mm and a slice-in-place resolution of 1.0 mm × 1.0 mm (repetition time [TR] = 2375 ms; echo time [TE] = 30 ms; flip angle = 8°; field of view [FOV] = 224 mm; acquisition matrix = 240 × 187).

#### fMRI Data Analysis

The raw data were preprocessed using the Data Processing Assistant for rs-fMRI (DPARSF 4.2) and the REST software on the MATLAB-R2012a platform. Those two pieces of software were often used in the resting-state analysis (Yan and Zang, 2010) to remove the effects of very-low-frequency drift and high-frequency noise such as respiratory and heart rhythms (Lowe et al., 1998). For each subject, we employed the slice timing to correct slice order, and then the data were realigned to estimate and modify head movement parameters; seven subjects with head movements that were >2.5 mm and >2.5° were excluded. After realignment, all data were normalized to the Montreal Neurological Institute (MNI) space. We discarded the first five images to ensure that all remaining images were magnet-steady, and the remaining 115 images were analyzed. The T1-weighted images were co-registered with the EPI mean images and segmented into white matter, gray matter, and cerebrospinal fluid (CSF). These images were then normalized to the MNI space in 3 mm × 3 mm × 3 mm voxel sizes. The normalized data were spatially smoothed with a Gaussian kernel; the full width at half maximum (FWHM) was specified as 6 mm × 6 mm × 6 mm. The linear drift was removed and data was filtered with a band-pass filter (0.01–0.08 Hz) to reduce low-frequency drift and high-frequency respiratory and cardiac noise (Biswal et al., 1995). Next, the filtered time series were transformed into a frequency domain with a fast Fourier transform (FFT) and the power spectrum was obtained. Finally, the square root at each frequency of the power spectrum was calculated and the averaged square root was obtained across 0.01–0.08 Hz at each voxel. This averaged square root was taken as the ALFF, which was then converted into the zALFF (Zang et al., 2007). The zALFF was used to compare spontaneous brain activity between the two groups of participants.

## Results

### Future Limitation Perspective

We examined group differences in future limitation perspective by running a two-sample *t*-test to verify the effectiveness of the grouping. The results indicated significant difference between the S-FLP group (*M* = 25.49, *SD* = 2.46) and W-FLP group (*M* = 15.41, *SD* = 1.46) in terms of limitation scores, *t*[70] = 23.88, *p* < 0.001. The findings indicate that the S-FLP had a significantly stronger sense of future limitations than did the W-FLP group.

### Fear of Cancer Recurrence

To examine the differences in fear of cancer recurrence, we performed a two-sample *t*-test between the S-FLP and W-FLP groups. The results showed that there was a significantly stronger subjective fear of cancer recurrence in the S-FLP group (*M* = 6.64, *SD* = 1.37) than in the W-FLP group (*M* = 3.94, *SD* = 0.92), *t*[76] = 11.15, *p* < 0.001. The results suggested that individuals with a stronger sense of future limitations had a stronger fear of disease recurrence.

### Mental Health Status

Ninety-one percent cancer patients chose the answer “Yes.” This result reflected that cancer is a main reason in causing a series of psychological changes.

To investigate the differences in MHS, we examined the group difference in the total score on the SCL-90-R using a two-sample *t*-test. The results showed that the S-FLP group (*M* = 193.36, *SD* = 33.73) had significantly stronger subjective psychological symptoms than that in the W-FLP group (*M* = 133.53, *SD* = 22.62), *t*[64] = 9.71, *p* < 0.001. Thus, those with a stronger FLP had more severe psychological symptoms.

### Resting-State MRI Results

**FIGURE 1:**
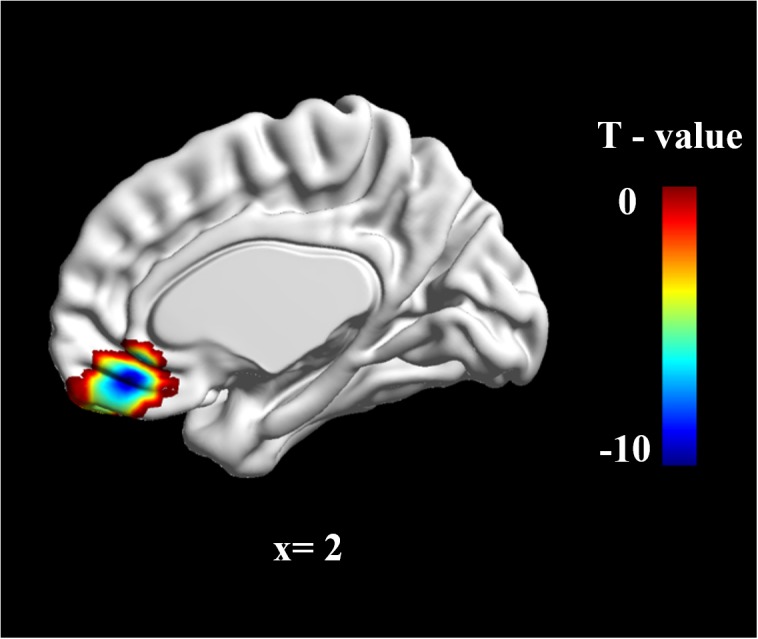
zALFF differences in vmPFC between S-FLP group and the W-FLP group (*p* < 0.01, Alphasim corrected; Voxels ≥ 40). There was much greater significant negative activation in S-FLP group than that in the W-FLP group.

To determine group differences in resting-state brain spontaneous activity, we performed a two-sample *t*-test. The results showed that there was much stronger brain activation in the vmPFC in the W-FLP group than in the S-FLP group [peak voxel coordinate, (3, 42, -15), *t*(92) = 9.75, Voxel-wise FDR-corrected, *p* = 0.05, *K* = 451, voxels ≥ 10; see **Figure 1**].

Region of interest (ROI) analysis was performed using two-sample *t*-tests for the difference in zALFF values between the S-FLP and W-FLP group. ROIs were selected on the basis of coordinates identified in a prior meta-analysis (*x*, *y*, *z* = 2, 40, -16) (Diekhof et al., 2011) with a radius of 6 mm (Milad et al., 2007). This analysis showed that the W-FLP group (*M* = 0.50, *SD* = 0.37) had significantly greater spontaneous brain activity in the vmPFC than that in the S-FLP group (*M* = -0.30, *SD* = 0.23), *t*[80] = -12.64, *p* < 0.001.

### Brain–Behavior Correlations Results

The FLP, FCR, vmPFC (zALFF), and SCL-90-R total scores significantly differed according to the *t*-test results. We further computed Pearson correlations among those variables for each group to determine whether there are any correlation tendencies among the variables in whole participants and two groups respectively (see **Table 1**). For all participants, the correlation analysis showed that the scores of FLP (*r* = -0.78, *p* < 0.01), FCR (*r* = -0.69, *p* < 0.01), and SCL-90-R total score (*r* = -0.82, *p* < 0.01) were negatively correlated with spontaneous activity in the vmPFC (zALFF). Furthermore, changes of the FCR (*r* = 0.77, *p* < 0.01) and SCL-90-R total score (*r* = 0.78, *p* < 0.01) were positively correlated with the FLP; changes in the FCR (*r* = 0.65, *p* < 0.01) were also positively correlated with the SCL-90-R total score. Moreover, the brain-behavior correlations results passed the multiple comparisons corrected (Alpha = 0.05/3 = 0.0167, the *p*-values of the correlation results between the brain data and the other three behavioral data were less than 0.0167). The results suggested that FLP, FCR, and MHS negatively correlated with spontaneous activity in the vmPFC (zALFF), and FLP, FCR and MHS positively correlated with each other in all participants.

**Table 1:** Means, standard deviations, and correlations.

Group	Variable	Mean	*SD*	FLP	FCR	SCL90
S-FLP	FLP	25.49	2.46	1		
	FCR	6.64	1.37	0.295*	1	
	SCL90-R	193.36	33.73	0.556***	0.418**	1
	vmPFC	-0.30	0.23	-0.341*	-0.783***	-0.674***
W-FLP	FLP	15.41	1.46	1		
	FCR	3.94	0.92	0.128	1	
	SCL90-R	133.53	22.62	0.072	-0.171	1
	vmPFC	0.50	0.37	-0.100	0.202	-0.621**
Both groups	FLP	20.23	5.44	1		
	FCR	5.23	1.78	0.768**	1	
	SCL90-R	162.17	41.29	0.780**	0.654**	1
	vmPFC	0.12	0.51	-0.778**	-0.690**	-0.821**


**N_(*S-FLP*)_* = 45, *N_(*W-FLP*)_* = 49, *N_(*Both groups*)_* = 94; ^∗^*p* < 0.05, ^∗∗^*p* < 0.01, ^∗∗∗^*p*< 0.001.*

Additionally, we also analyzed the correlation data of S-FLP and W-FLP groups respectively (see **Table 1**). The results suggested that stronger FLP and higher scores of FCR and SCL-90-R lead to the activity of vmPFC to be lower, and despite weaker FLP, there was no significant correlation between FCR and SCL-90-R, but there was a relatively higher activity of vmPFC.

### Path Analysis Results

**FIGURE 2:**
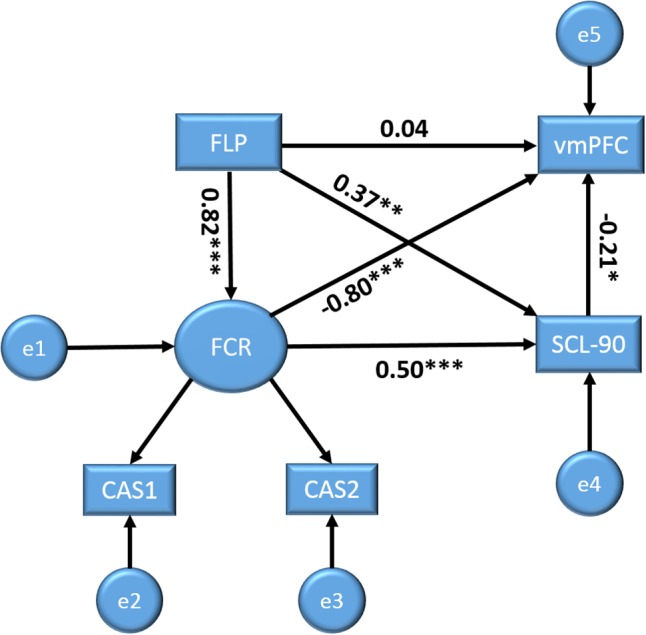
Structural equation models with future Limitation perspective (FLP), fear of cancer recurrence (FCR), and MHS as predictors for brain activity (vmPFC) outcome variables. Displayed values are standardized regression weights. ^∗^*p* < 0.05, ^∗∗^*p* < 0.01, ^∗∗∗^*p* < 0.001.

Based on prior research and the above analyses, we further elucidated the relationships among the study variables using structural equation modeling (SEM). In the model, we set FLP, FCR, and MHS as predictors of brain activity (zALFF). The SEM was conducted using SPSS AMOS 21.0. The model showed an adequate fit to the data, χ^2^(2) = 2.74, Probability level = 0.26, normed fit index = 0.99, relative fit index = 0.97, incremental fit index = 0.99, Tucker–Lewis index = 0.99, comparative fit index = 0.99, root mean square error of approximation = 0.06, standardized root mean square residual = 0.01. **Figure 2** shows the corresponding model with the standardized regression weights (SRW). FLP had no significant direct path to vmPFC activity, SRW = 0.04, *p* = 0.72, but did have a significant direct path to FCR, SRW = 0.82, *p* < 0.001, and MHS, SRW = 0.37, *p* < 0.01. Notably, as the absolute value of the standardized direct effects (0.04) was less than the absolute value of the standardized indirect effects (-0.81) via FCR and MHS, these variables can completely mediate the relationships between FLP and vmPFC activities. Furthermore, FCR directly related to vmPFC activity, SRW = -0.80, *p* < 0.001, as did MHS, SRW = -0.21, *p* < 0.05. FCR also was directly related to MHS, SRW s = 0.50, *p* < 0.001. The model showed that FLP significantly related to vmPFC activities via the mediation variables FCR and MHS (see **Figure 2**).

## Discussion

The main objective of the current study was to determine the relationships among FLP, FCR, MHS, and brain activity. More specifically, we first found that FLP was a substantial predictor of FCR–that is, the S-FLP group reported higher FCR than did the W-FLP group. Although both groups had been treated for over a year and the average age was about 50 years old, the S-FLP group had a greater tendency to adjust their subjective future perspective to be closer to their time of death and believed that they had little remaining lifetime. Therefore, they exhibited a greater fear of the future and a more negative attitude toward the treatment of their disease, constantly worrying about disease recurrence. Their greater perceived time limitation might have made them more sensitive to the changes in health status, which further causes them to be more worried about cancer recurrence. By contrast, the W-FLP group not only had lower FLP, but also worried less about the recurrence of cancer than the S-FLP group. According to terror-management theory (Burke et al., 2010), mortality salience increased the feelings of FLP, which is the mechanism of FCR. As the time of treatment is prolonged the patient’s understanding of the disease and treatment program gradually increases, and they begin to generate fear of cancer recurrence and metastasis. But different levels of FLP affect the degree of fear of cancer recurrence in the patient. FLP could be affected by different personal factors such as behavioral attitudes (Rotenberg, 2009) toward cancer, sense of coherence and dispositional optimism (Gilbar, 2003). These factors may lead to different levels of FLP, which further leads to different degrees of FCR. The fear of cancer recurrence might in turn affect FLP.

Second, S-FLP produced greater FCR; meanwhile both FLP and FCR were together positive predictors of MHS. Previous studies have also shown that FCR can lead to problematic psychological behaviors, including anxious preoccupation, depression, avoidance, and excessive checking, contributing to an increased fear response (van den Beuken-van Everdingen et al., 2008). Patients with S-FLP and high FCR may tend to be vulnerable to mental disorders, perhaps due to their enhanced sensitivity to aversive stimuli or lacking the ability to deal with negative stimuli (Custers et al., 2015). Although the W-FLP group also exhibited psychological symptoms, their mental adjustment ability (defined as ‘cognitive and behavioral responses made by an individual’) appears to be better than that of the S-FLP group (Greer et al., 1989). In psychosocial research on cancer patients, mental adjustment is a widely studied concept. Many studies have suggested that the mental adjustment ability of cancer patients is one of the most important factors influencing quality of life and degree of psychological distress (Watson et al., 1991). Therefore, our results suggest that the S-FLP group’s mental adjustment system maybe dysfunctional. This might be related to changes in brain mechanisms, as discussed below.

Third, the neuroimaging results indicated that the vmPFC activity was diminished in the S-FLP group relative to the W-FLP group. Numerous previous studies have indicated that the vmPFC might represent a domain-general controller of perceived fear and aversion that modulates the negative affective responses of the emotion processing system (Diekhof et al., 2011; Lin et al., 2016). When the activity of vmPFC is enhancement, negative affective responses can be better controlled and the degree of unpleasantness of events might be reduced. Therefore, we infer that most cancer patients had a variety of negative emotions and psychological symptoms at the beginning stage of their cancer diagnosis, but some cancer patients continued to show high levels of negative emotion and psychological symptoms and others returned to normal levels as their treatment progressed. This speculation indicates that long-term high levels of negative emotion and psychological symptoms probably led to dysfunction in the vmPFC. Another inference was that before the S-FLP group got cancer, their vmPFC was already dysfunction, and which manifested as psychological and emotional regulation dysfunction, and further developed a relatively stable unhealthy state of mind. Due to the fact that this study is not a longitudinal study, the causal relationship between these variables cannot be clearly explained. Although the result that there was difference in MHS before getting cancer cannot directly reflect the corresponding condition of vmPFC, at least cancer has brought a series of changes. So in this study, we did not consider the condition that the vmPFC was dysfunctional before getting cancer. We were trying to construct the path analysis which was based on the correlation analysis of the four variables and the theoretical framework mentioned above to further test the direction and magnitude of the direct effect of independent variables on dependent variables.

Fourth, the path analysis showed that FLP did not directly predict vmPFC activity, but rather indirectly via FCR and MHS. Therefore, we speculated that when people learn that they have cancer the first intuitive psychological experience is that their future is limited (Bölter et al., 2010), and then FLP produces many sorts of negative emotions, including fear that the disease cannot be cured and that it may cause death. Then a variety of psychological disorders were created. Suffering mental problems and fear might decrease activity of vmPFC. This process of changing brain function does not happen instantaneously, and the decrease in vmPFC activity is caused by a long period of negative stimulation. Therefore, this model suggests that although vmPFC’s function is the top-down regulation of emotion and behavior (Etkin et al., 2011), the reverse is also susceptible to long-term abnormal mental states and negative emotions.

### Limitations and Future Studies

This study has several limitations. First, this study is cross-sectional, and therefore cannot explore the causal relationships among FLP, mental health, fear, and brain activity; future research can be applied to longitudinal, in-depth study of the change of FLP effect on mental health and brain function. Second, as for why some people are prone to form S-FLP and change in brain function, we did not examine these possible causal factors such as personality, genes, sense of coherence, dispositional optimism (Gilbar, 2003), and behavioral attitudes (Rotenberg, 2009) toward cancer in this study. For instance, “Different behavioral attitudes may determine the state of mental health because giving up is strong related to depression and destructive anxiety. Giving up as a general behavioral attitude can explain enhanced sensitivity to aversive stimuli and decrease the ability to deal with negative experiences.” Thus, further studies on the issue may be recommended to provide conclusive outcomes.

### Clinical Implications

The present study provides a new perspective preliminarily constructed a relationship model among time perspective, mental problems, and brain activity in cancer patients. This study has important clinical implications in both developing clinical counseling strategies to adjust the patient’s time perspective further to alleviate the strong life limitation of cancer patients, and suggesting that the clinical doctors pay more attention to the changes of brain area vmPFC activity.

## Conclusion

This study indicates that a strong sense of FLP can lead to severe mental problems and fear about the recurrence of cancer. Furthermore, excessive fear appears to exacerbate increased psychological symptoms, and both may lead to changes in vmPFC activity. The vmPFC might play a key role in dealing with negative events. Dysfunction of the vmPFC, in turn, may aggravate the mental distress of cancer patients, causing some cancer patients to be unable to regulate their own mental disorders.

## Author Contributions

JZ designed and conducted the experiment protocol, analyzed the data, and drafted this manuscript; XL, XH, and YY participated in conducting experiments; PF, GJ, and JS participated in the development of the encoding principles and reviewed the manuscript; YZ reviewed the manuscript and provided important comments and revision. All authors approved the final manuscript.

## Conflict of Interest Statement

The authors declare that the research was conducted in the absence of any commercial or financial relationships that could be construed as a potential conflict of interest.
